# ‘Artilysation’ of endolysin λSa2lys strongly improves its enzymatic and antibacterial activity against streptococci

**DOI:** 10.1038/srep35382

**Published:** 2016-10-24

**Authors:** Lorena Rodríguez-Rubio, Wai-Ling Chang, Diana Gutiérrez, Rob Lavigne, Beatriz Martínez, Ana Rodríguez, Sander K. Govers, Abram Aertsen, Christine Hirl, Manfred Biebl, Yves Briers, Pilar García

**Affiliations:** 1 Instituto de Productos Lácteos de Asturias (IPLA-CSIC), Paseo Río Linares s/n 33300- Villaviciosa, Asturias, Spain; 2 Laboratory of Gene Technology, Department of Biosystems, KU Leuven, Kasteelpark Arenberg 21 – box 2462, 3001 Heverlee, Belgium; 3 Lisando GmbH, Am BioPark 13, 93053 Regensburg, Germany; 4 Laboratory of Food Microbiology, Department of Microbial and Molecular Systems, KU Leuven, Heverlee, Belgium; 5 Laboratory of Applied Biotechnology, Department of Applied Biosciences, Ghent University, Ghent Belgium

## Abstract

Endolysins constitute a promising class of antibacterials against Gram-positive bacteria. Recently, endolysins have been engineered with selected peptides to obtain a new generation of lytic proteins, Artilysins, with specific activity against Gram-negative bacteria. Here, we demonstrate that artilysation can also be used to enhance the antibacterial activity of endolysins against Gram-positive bacteria and to reduce the dependence on external conditions. Art-240, a chimeric protein of the anti-streptococcal endolysin λSa2lys and the polycationic peptide PCNP, shows a similar species specificity as the parental endolysin, but the bactericidal activity against streptococci increases and is less affected by elevated NaCl concentrations and pH variations. Time-kill experiments and time-lapse microscopy demonstrate that the killing rate of Art-240 is approximately two-fold higher compared to wildtype endolysin λSa2lys, with a reduction in viable bacteria of 3 log units after 10 min. In addition, lower doses of Art-240 are required to achieve the same bactericidal effect.

Endolysins are bacteriophage-encoded muralytic enzymes. They digest the peptidoglycan at the last stage of the lytic cycle, resulting in the release of newly formed virions from the infected host bacteria. Endolysin efficacy is of crucial importance for a successful bacteriophage multiplication cycle. Therefore, throughout evolution, bacteriophages have acquired highly efficient enzymes featured by high specificity^1^ and a low probability of developing bacterial resistance^2^,^3^,^4^. Endolysins are composed by a single or multiple catalytic domains and in many of them a cell wall binding domain is present^5^. This feature has facilitated endolysin engineering to design new chimeric proteins with desired properties like altered specificity^6^ and enhanced lytic activity^7^,^8^.

The finding that endolysins are able to attack Gram-positive bacteria and lyse them when added exogenously prompted their study as novel antimicrobial agents. Numerous *in vitro* and *in vivo* studies have shown the efficacy of endolysins to eliminate pathogenic bacteria from mucosal surfaces and systemic infections^9^. These characteristics, along with their efficacy against antibiotic-resistant bacteria, lack of toxicity and synergy with antibiotics makes them promising candidates to be exploited in the therapy of infectious diseases. In addition to clinical use, other applications of endolysins include their use in food safety, pathogen detection, disinfection, nanotechnology and vaccine development^10^.

Recently, endolysins have been engineered to overcome one of the main limitations in their use, i.e., their inefficacy against Gram-negative bacteria due to the presence of the outer membrane. Artilysins are optimized fusions of selected endolysins to specific outer membrane permeabilizing peptides, which promote transfer of the fusion protein across the outer membrane structure^11^. Upon translocation, the peptidoglycan is degraded, causing a rapid cell death by osmotic lysis^12^. Artilysins have been developed against Gram-negative pathogens including *Pseudomonas aeruginosa* and *Acinetobacter baumannii in vitro* and *in vivo*, and have a low probability of resistance development similar to endolysins. They are highly bactericidal against multidrug-resistant isolates and persisters and may therefore be useful in the treatment of human and animal chronic infections^11^,^13^,^14^.

The endolysin λSa2lys from *Streptococcus agalactiae* prophage λSa2 has two catalytic domains showing γ-D-glutaminyl-L-lysine endopeptidase and β-D-N-acetylglucosaminidase activity, respectively, and one cell wall binding domain (CBD), which comprises on its turn two Cpl-7 type subdomains^15^. This enzyme has lytic activity against *Streptococcus pyogenes*, *Streptococcus dysgalactiae*, *Streptococcus uberis*, *Streptococcus equi*, Group G *Streptococcus* and Group E *Streptococcus*^16^. Chimeric proteins derived from endolysin λSa2lys through fusion of the endopeptidase domain to staphylococcal cell wall binding domains from either lysostaphin or staphylococcal endolysin LysK are highly active against *Streptococcus agalactiae*, *S. uberis* and *Staphylococcus aureus*^17^,^18^.

In this study, the ‘artilysation’ of an endolysin, i.e. the modification of an endolysin with a specific peptide with a dedicated function, was explored as a new approach to improve the muralytic and antibacterial properties of endolysins acting against Gram-positive bacteria. Contrary to Gram-negative targeting Artilysins, which comprise a peptide with an outer membrane permeabilizing function, the peptide in this novel Artilysin targeting a Gram-positive species is selected to improve cell wall affinity under a broader range of environmental conditions such as pH and ionic strength. As such, ‘artilysation’ does not only unlock the antibacterial potential of endolysins against Gram-negative species, but would also strengthen the antibacterial activity against Gram-positive species in a broader scope of applications. Here, we report on Art-240, a chimeric endolysin which is a fusion of the polycationic nonapeptide (PCNP) (composed of a mixture of arginine and lysine residues^11^) to the C-terminus of λSa2lys. Analysis of Art-240 shows that artilysation generally improves both the muralytic and antibacterial properties of λSa2lys under a broad range of conditions.

## Results

### Art-240 has the same specificity as λSa2lys, but shows increased bactericidal activity

Both λSa2lys and Art-240 were purified to homogeneity by metal affinity chromatography, resulting in a total yield of 11.5 and 8.36 mg/l of *Escherichia coli* expression culture, respectively. Purity of proteins was evaluated by SDS-PAGE (>95%). Major protein bands with estimated molecular masses of about 50 kDa, correlating well to the predicted molecular masses for λSa2lys and Art-240 (50.8 and 53.4 kDa), were observed (Supplementary File 1A,B). Melting curves of both λSa2lys and Art-240 were determined by monitoring the loss of α-helicity by CD spectroscopy at 222 nm and show that the structural stability of λSa2lys is conserved after artilysation with melting temperatures of 61.1 °C and 63.9 °C, respectively (Supplementary File 1C).

The specificity of the antibacterial activity of Art-240 and endolysin λSa2lys was initially compared using a plate lysis assay against a collection of thirty-nine Gram-positive and five Gram-negative bacterial strains (Table 1). The activity spectrum is similar for both proteins at the highest concentration tested (5 μM). Indeed, all streptococcal species tested (*S. agalactiae*, *S. dysgalactiae*, *S. pyogenes*, *S. uberis*, *S. suis*, *S. porcinus*, *S. gordonii*, *S. sanguinis*, and *S. viridans*) and one *Staphylococcus epidermidis* strain are sensitive, whereas other Gram-positive species such as *S. aureus* and *Enterococcus faecium* are not affected. Artilysation does not expand the lytic activity of endolysin λSa2lys to Gram-negative *P. aeruginosa*, *E. coli* and *Salmonella* strains. However, the antibacterial activity is clearly improved for the majority of sensitive strains in case of Art-240, as lower concentrations of the protein are required to observe a similar lysis spot (Supplementary File 2).

**Figure 1:**
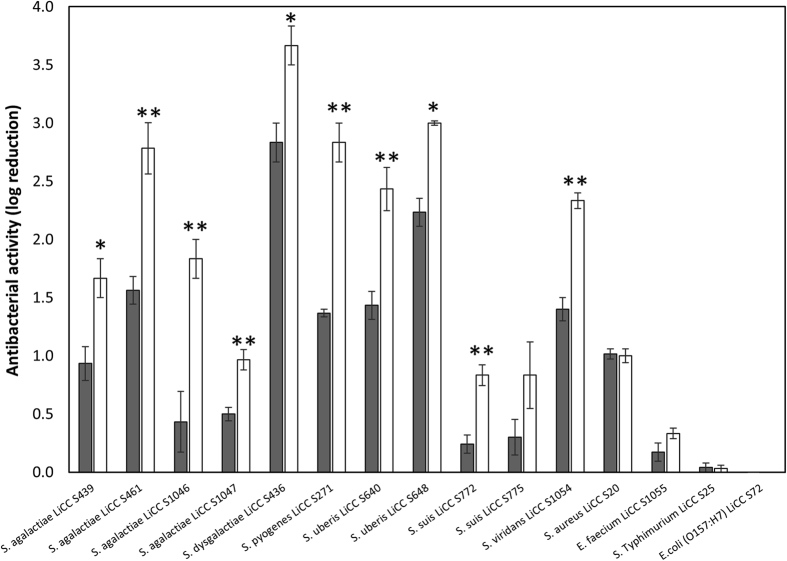
Bactericidal effects of endolysin λSa2lys and Art-240 against different bacterial species. A cell suspension of several streptococcal species, *S. aureus* LiCC S20, *E. faecium* LiCC S1055 *Salmonella* Typhimurium LiCC LiCC S25 and *E. coli* (O157:H7) LiCC S72 were treated with equimolar amounts (0.1 μM) of λSa2lys (grey) endolysin and Art-240 (white). Bacterial reduction after 1 h is expressed in log units relative to the untreated control. Data reported are means ± standard deviations of three replicates. Student *t*-test was performed to compare the bactericidal activity of λSa2lys and Art-240. *P < 0.05; **P < 0.01.

These observations were quantified using killing assays on a panel of 15 strains (four *S. agalactiae*, one *S. dysgalactiae*, one *S. pyogenes*, two *S. uberis*, two *S. suis*, one *S. viridans*, one *S. aureus*, one *E. faecium*, one *S.* Typhimurium and one *E. coli* O157:H7). Cell suspensions were treated with equimolar amounts (0.1 μM) of both proteins and cell number reductions were analyzed after an exposure of 1 h at 25 °C. Artilysation notably increases the antibacterial activity against most of the tested strains, causing a reduction of bacterial viability by 0.5–1.5 log units higher than endolysin λSa2lys (Fig. 1). For instance, Art-240 reduced the cell number of *S. agalactiae* LiCC S461 by 2.78 ± 0.38 log units whereas a reduction of 1.56 ± 0.21 log units was detected after λSa2lys treatment (*P* < 0.01); a reduction of 2.83 ± 0.29 log units in cell number was observed for *S. pyogenes* LiCC S271 compared to 1.37 ± 0.06 log units after λSa2lys treatment (*P* < 0.01), and 2.43 ± 0.32 log units compared to 1.43 ± 0.21 log units in case of *S. uberis* LiCC S640 (*P* < 0.01). The effect of Art-240 and λSa2lys was similar on *S. aureus* LiCC S20 (*P* > 0.05). Notably, the bactericidal effect against *S. aureus* shows the higher sensitivity of the killing assay compared to the plate lysis assay where no inhibition of staphylococcal strains was observed. *E. faecium*, *S.* Typhimurium and *E. coli* species were not significantly affected by Art-240.

### Art-240 has increased enzymatic activity over a broader pH range and a broader range of salt concentration

**Figure 2:**
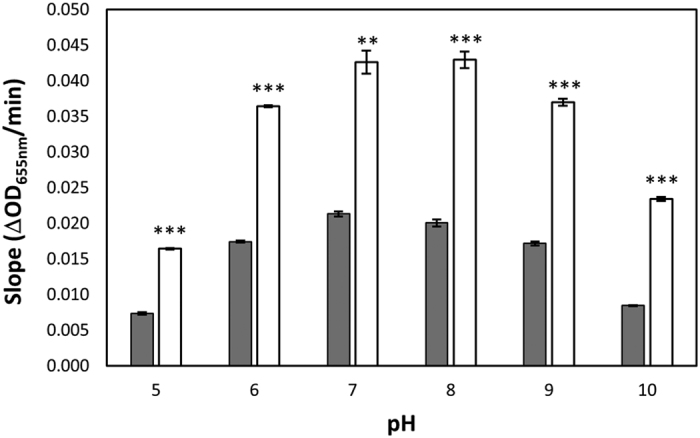
Effect of pH on the enzymatic activity of endolysin λSa2lys and Art-240. The enzymatic activities were determined using a turbidity reduction assay with equimolar amounts (0.1 μM) of λSa2lys (grey) and Art-240 (white), and reported as the slopes derived from 60-min curves from *S. agalactiae* LiCC S461 in the exponential phase performed under various pH conditions. Data are means ± standard deviations of three replicates. Student *t*-test was performed to compare the activity of λSa2lys and Art-240 within each pH value. **P < 0.01: ***P < 0.001.

The muralytic activity of Art-240 against *S. uberis* and *S. agalactiae* was tested at different pH values and compared to the activity of the λSa2lys endolysin. Enzymatic activity of Art-240 against *S. agalactiae* LiCC S461 (Fig. 2) is higher than that of λSa2lys at all assayed pH values (*P* < 0.01; *P* < 0.001). Similar to endolysin λSa2lys, Art-240 shows an optimal activity at pH 7–8. When *S. uberis* LiCC S648 is used as substrate (Supplementary File 3), a similar outcome is observed, but with more pronounced differences for alkaline pH values. A >6-fold increase in enzymatic activity is observed for Art-240 at pH 9 (*P* < 0.001). Art-240 retains some activity at pH 10, contrary to λSa2lys. In summary, artilysation of λSa2lys improves the enzymatic activity in a broad pH range.

**Figure 3:**
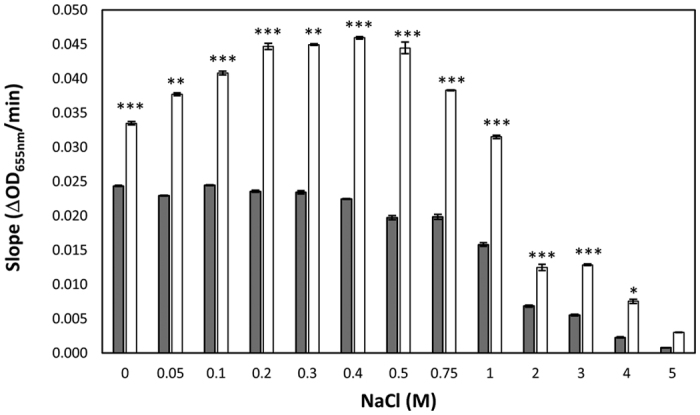
Effect of NaCl on the enzymatic activity of endolysin λSa2lys and Art-240. The enzymatic activities were determined by using a turbidity reduction assay using equimolar amounts (0.1 μM) of λSa2lys (dark) and Art-240 (white), and reported as the slopes derived from a 60-min curves from *S. agalactiae* LiCC S461 in the exponential phase performed under various sodium chloride (NaCl) conditions. Data are means ± standard deviations of three replicates. Student *t*-test was performed to compare the activity of λSa2lys and Art-240 within each NaCl concentration. **P < 0.01; ***P < 0.001.

The effect of artilysation was evaluated with regard to salt tolerance for both *S. agalactiae* LiCC S461 (Fig. 3) and *S. uberis* LiCC S648 (Supplementary File 4). The enzymatic activity of Art-240 is significantly higher than the activity of λSa2lys over a broad range of NaCl concentrations for both *S. agalactiae* LiCC S461 (*P* < 0.01; *P* < 0.001) and *S. uberis* LiCC S648 (*P* < 0.01; *P* < 0.001). Although the enzymatic activity of Art-240 is also clearly affected by NaCl concentrations above 0.5 M (*S. agalactiae* LiCC S461) or 0.75 M (*S. uberis* LiCC S648), activity could be detected at high salt conditions up to 5 M NaCl. Artilysation with a polycationic peptide leads thus to increased enzymatic activity under a broad range of NaCl concentrations.

### Art-240 is enzymatically active against both exponential and stationary bacterial cells

**Figure 4:**
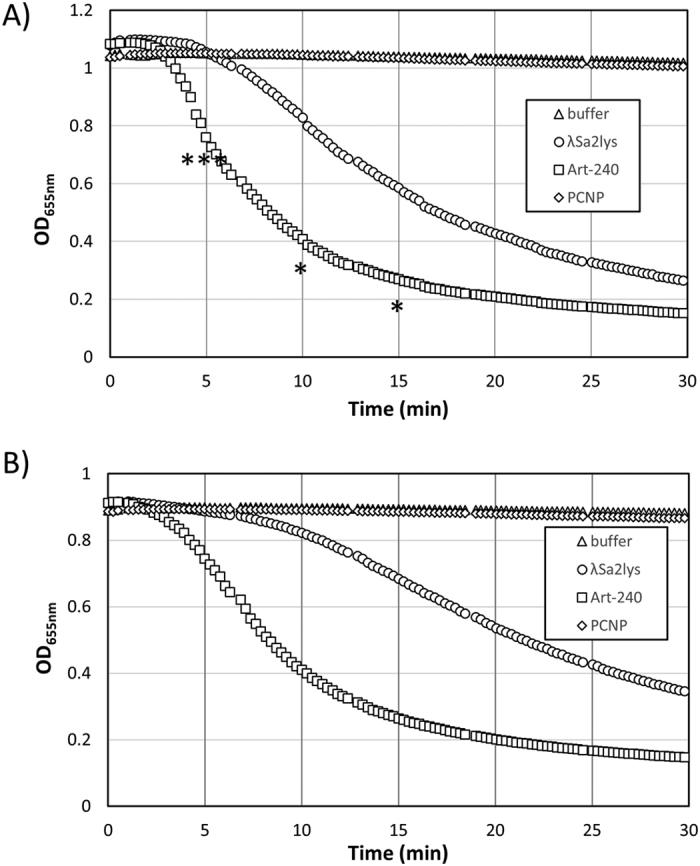
Activity of endolysin λSa2lys and Art-240 on both exponential and stationary *S. agalactiae* LiCC S461. Turbidity reduction assay with equimolar amounts (0.1 μM) of λSa2lys and Art-240 are shown for both mid-exponential phase (**A**) and stationary phase (**B**) *S. agalactiae* LiCC S461 cells. Data are means ± standard deviations of three independent experiments. Student *t*-test was performed to compare the activity of λSa2lys and Art-240 within each growth phase. *P < 0.05; ***P < 0.001.

To analyze the influence of the growth stage on the susceptibility to Art-240, turbidity reduction assays using 0.1 μM of both proteins (Art-240 and endolysin λSa2lys) have been performed. *S. agalactiae* LiCC S461 cells collected during exponential (Fig. 4A) and stationary (Fig. 4B) growth phase are both susceptible to Art-240 with an enzymatic activity of 121 ± 16 and 84 ± 27 units, respectively. Similarly, endolysin λSa2lys is also active against cells in both growth stages but with reduced activities (61 ± 13 and 30 ± 4 units against exponential and stationary cells, respectively). Significant differences could be observed between Art-240 and endolysin λSa2lys activities on exponential cells after 5, 10 and 15 min of treatment (*P* < 0.05; *P* < 0.001). The polycationic nonapeptide (PCNP) alone or the buffer alone do not lyse cells in both phases. Stationary phase cells are thus less susceptible for λSa2lys and Art-240, but the reduced activity of Art-240 against stationary phase cells remains similar compared to the enzymatic activity of λSa2lys against exponential phase cells. Artilysation of λSa2lys thus compensates the reduction in activity which is intrinsically related to stationary phase cells.

### Art-240 kills bacteria faster than its parental endolysin λSa2lys

**Figure 5:**
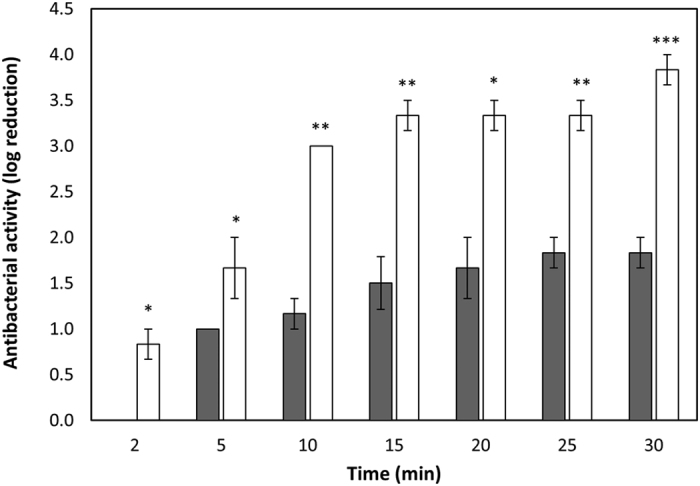
Rapid mode of action of Art-240. Time-kill experiment of *S. agalactiae* LiCC S461 treated with 0.1 μM Art-240 (white) and endolysin λSa2lys (grey) at pH 7.4. Results (means ± standard deviations of three replicates) are reported as bacterial reduction (log_10_ units) relative to the untreated control. Student *t*-test was performed to compare the activity of λSa2lys and Art-240. *P < 0.05; **P < 0.01; ***P < 0.001.

**Figure 6:**
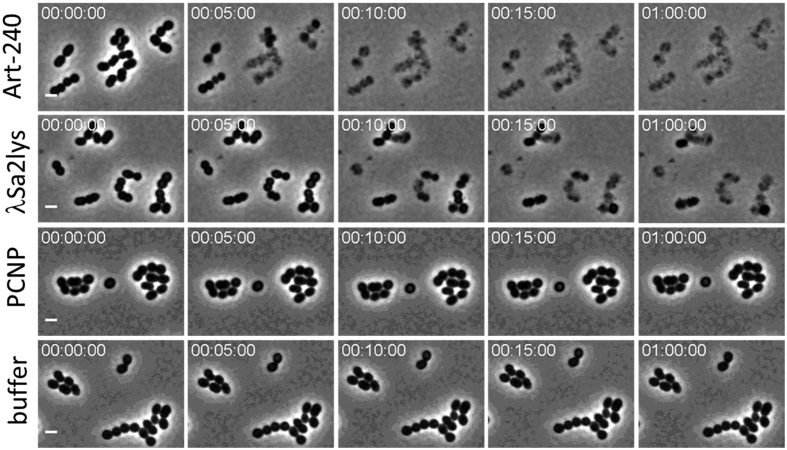
Real-time time lapse series of *S. uberis* LiCC S648 exposed to equimolar amounts (0.1 μM) of Art-240, λSa2lys, PCNP and buffer. Exponentially growing cells were washed three times with buffer and subsequently mixed (1:1) with 0.2 μM of the corresponding enzyme/peptide. The mixture was dropped on agarose pads and cells were monitored over 1 h in real-time (Supplementary Movie S1). Five minute intervals are shown for each condition during the first 15 minutes and after 1 h. Scale bar = 2 μm.

To quantify the killing rate of Art-240 time-kill experiments were performed using *S. agalactiae* LiCC S461 cultures treated with 0.1 μM of each lytic protein at the optimal pH (pH 7) (Fig. 5). Art-240 acts more rapidly than λSa2lys. Art-240 causes a reduction in cell number of 1.7 ± 0.3 log units after 5 min (*P* < 0.05), whereas λSa2lys needs 30 minutes to cause a similar reduction in cell number (1.8 ± 0.1 log units). The rapid onset of action by Art-240 is also evidenced after 2 min of incubation (*P* < 0.05) with a reduction in the viable bacterial count (one log unit) while no activity of the endolysin λSa2lys is detected after this short exposure time. In addition, the extent of killing is significantly greater in case of Art-240 with a reduction of 3.8 ± 0.2 log units after 30 min. This represents a twofold increase compared to λSa2lys at the same time point (*P* < 0.001). The experiment was repeated under identical conditions, but now time-dependent killing was monitored using time-lapse microscopy (Fig. 6, Movie S1). While both λSa2lys and Art-240 induce osmotic lysis of bacterial cells, the more rapid killing of Art-240 is clearly visualized and a more complete lysis is achieved in comparison to the λSa2lys endolysin. Controls (PCNP or buffer alone) do not visibly affect the structural integrity.

### Lower doses of Art-240 are required to achieve a similar bactericidal effect against *S. agalactiae* LiCC S461 compared to λSa2lys

**Figure 7:**
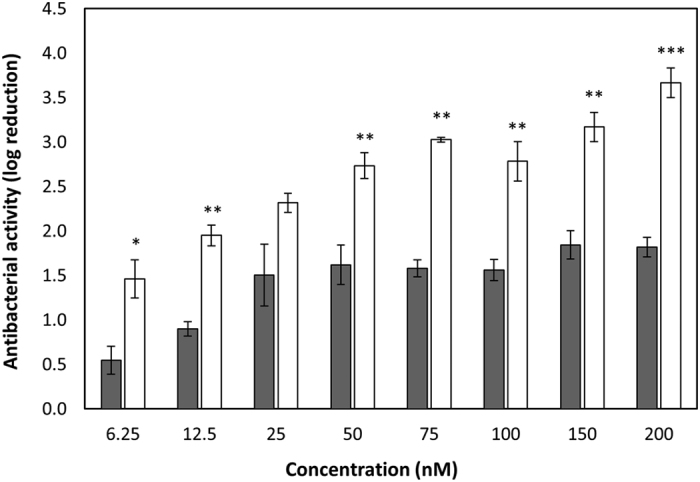
Art-240 kills *S. agalactiae* LiCC S461 in a rapid and efficient way. A cell suspension at pH 7.4 was treated with increasing amounts of λSa2lys (grey) and Art-240 (white). Antibacterial activity was reported as bacterial reduction (log_10_ units) relative to the untreated control after 1 h of treatment. Data are means ± standard deviations of three replicates. Student *t*-test was performed to compare the activity of λSa2lys and Art-240. *P < 0.05; **P < 0.01.

A dose-response curve from 6.25 nM to 200 nM of Art-240 was determined at pH 7. The bactericidal effect was quantified after 1 h of incubation for the different doses and compared to those obtained after endolysin λSa2lys exposure (Fig. 7). The antibacterial activity of Art-240 outcompetes the λSa2lys antibacterial activity, which is most outspoken at low concentrations (*P* < 0.05). To achieve the same bactericidal effect, a 4 to 12-fold higher dose of λSa2lys is needed. E.g., a reduction of about 2 log units is observed for 12.5 nM Art-240, while 150 nM λSa2lys is needed to cause the same reduction. Moreover, an increase in λSa2lys concentration over 50 nM does not further improve the antibacterial activity but a higher reduction in bacterial counts (*P* < 0.01; *P* < 0.001) is achieved with Art-240 (Fig. 7). In conclusion, Art-240 is more effective and requires smaller doses than the endolysin to achieve a comparable bactericidal effect.

## Discussion

Artilysins represent a new class of engineered endolysins with high antibacterial activity against Gram-negative bacteria. The design of these enzymes implies the fusion of a selected outer membrane permeabilizing peptide to a specific endolysin. The cationic or amphipathic peptide moiety destabilizes the lipopolysaccharide (LPS) layer of the outer membrane by interfering with the ionic interactions between divalent cations and LPS phosphate groups and the hydrophobic forces between the fatty acids of lipid A that stabilize the outer membrane structure^12^. The peptidoglycan layer becomes accessible to the endolysin moiety, resulting in peptidoglycan degradation and cell death by osmotic lysis. Here, we have shown that the process of artilysation, i.e. modifying the properties of an endolysin by fusion with a specific peptide, represents a novel approach to improve the enzymatic and antibacterial properties of an endolysin active against Gram-positive bacteria. For Art-240 we made use of the polycationic nonapeptide (PCNP) which was previously successfully used in the construction of Artilysins against Gram-negative *P. aeruginosa*. The rationally designed Art-240 outperforms λSa2lys, the endolysin from which it is derived, in (i) an increased enzymatic activity under a broad range of pH and NaCl conditions, (ii) a higher overall bactericidal effect, (iii) a faster and more complete lysis/killing and (iv) the requirement of reduced doses. At the same time, the good protein stability of λSa2lys, its high specificity for streptococci and activity against stationary cells is conserved after artilysation.

Generally, endolysins are specific to a species (or genus) of bacteria naturally infected by the phages that encode them^19^. In good agreement with this, λSa2lys shows high activity against different species of *Streptococcus* and low activity against *S. epidermidis*^16^. Table 1 shows a similar specificity for Art-240, demonstrating that the PCNP did not extend the spectrum to other Gram-positive microorganisms, such as *E. faecium*, or Gram-negative bacteria. Indeed, the fusion of PCNP to endolysin λSa2lys may allow transport of Art-240 through the outer membrane of Gram-negative bacteria as has been shown before for other engineered endolysins^11^ but if this were to happen, the transfer appears not to be sufficient to lyse them. This was likely because of the different peptidoglycan chemotype between Gram-negative bacteria (A1γ) and streptococci (A3α)^20^. Indeed, the specificity of endolysins is due to the cell wall binding and/or the catalytic domains. λSa2lys harbors apart from two catalytic domains (a glucosaminidase and endopeptidase domain) a cell wall binding domain (CBD) that comprises two Cpl-7 subdomains^15^. Typically, CBDs of endolysins show a high affinity and specificity for their targets with low affinity constants^21^,^22^. Changes in the activity spectrum of the endolysin λSa2lys were previously described by deletion of its glucosaminidase domain. The truncated version harboring the endopeptidase and the CBD shows weak lytic activity against *S. aureus*, coagulase-negative staphylococci and *S. xylosus*^16^. Moreover, the specificity of the endolysin λSa2lys changes slightly by substituting the Cpl-7 cell wall binding domain for the staphylococcal-specific SH3b domain, rendering the protein active against both staphylococci and streptococci^7^.

We used both a plate lysis and killing assay and noted a different susceptibility for some strains, especially when the susceptibility is close to the detection limit. Specifically, *S. aureus* showed susceptibility in the killing assay, whereas no inhibition was observed in the plate lysis assay. This is consistent with previous reports stating that results from different assays are not always comparable^23^. Here, we believe that diffusion in the agar compromises the sensitivity of the plate lysis assay compared to the killing assay which was performed in suspension.

Overall, our results demonstrate that artilysation with a C-terminal PCNP increases both muralytic and antibacterial activity of λSa2lys. Based on our current knowledge, these improvements can be best explained by the positive charges of PCNP, which are expected to strengthen the interactions of Art-240 with the polyanionic cell surface (phosphate groups in teichoic acids) and consequently increases the local concentration at the site of action. The peptide PCNP (pI 12.3) solely contains positively charged arginines and lysines at pH 7.4. This increased contact of the catalytic domains of the endolysin with peptidoglycan may accelerate hydrolysis and subsequent osmotic lysis of the bacterial cell. In this regard, one may argue that the increased enzymatic activity of Art-240 over a broad pH range is also due to the highly positive charge of PCNP, because Art-240 (pI 8.8) is expected to interact with the bacterial surface in a broader pH range with a higher affinity compared to endolysin λSa2lys (pI 6.3) alone. A positive correlation between the positive charge of catalytic domains and bacteriolytic activity in the absence of a cell wall binding domain was previously described^24^. Moreover, an improved antibacterial activity was obtained by the inversion of the net charge (from negative to positive) of the cell wall binding domain of the Cpl-7 endolysin^25^. The hypothesis that electrostatic interactions constitute the basis for the enhanced antibacterial and muralytic activity of Art-240 compared to λSa2lys, is also supported by the ability of Art-240 to degrade peptidoglycan more efficiently than λSa2lys in a wide range of NaCl concentrations (0–5 M). The influence of ionic strength on the lytic activity of endolysins has been described previously and for most endolysins optimal activity was obtained at lower salt concentrations^26^,^27^,^28^,^29^. In the case of Art-240, the strong positive charge of the cationic peptide enhances the ability to remain bound to the cell wall in the presence of a high concentration of salt ions, which generally compete with electrostatic interactions.

In summary, this study demonstrates that artilysation is a promising strategy for the development of improved endolysin-based antimicrobials against Gram-positive bacteria. A more in-depth knowledge about the interactions between the endolysin and the peptide moieties of the Artilysin, and the peptide with the cell surface will help in the design of novel endolysins with enhanced antibacterial properties against any possible pathogen.

## Materials and Methods

### Bacterial strains and growth conditions

All bacterial strains used in this study were provided by Goldberg-Klinik Kelheim GmbH, Germany, RWTH University Aachen, Germany, Bayerisches Landesamtf. Gesundheit u. Lebensmittelsicherheit (LGL), Germany, Universitätsklinikum Regensburg, Germany, DSMZ (German Collection of Microorganisms and Cell Cultures), KU Leuven (Belgium), University of Veterinary Medicine Hanover (TiHo, Germany), or Robert Koch Institute (Wernigerode, Germany), each of which has been assigned with a specific LiCC (Lisando Culture Collection) number. All strains were grown in brain heart infusion broth (BHI) (Oxoid Deutschland GmbH), except *E. faecalis*, *S. aureus*, *E. coli* and *S.* Typhimurium, which were grown in lysogeny broth (LB; Sambrook *et al*., 1989) with or without shaking at 37 °C. *E. faecalis*, *S. suis, S. viridans* group, *S. sanguinis,* and *S. gordinii* were grown under microaerophilic conditions (85% N_2_, 10% CO_2_, 5% O_2_). Likewise, different *E. coli* strains were used in this study: *E. coli* NEB Turbo (New England Biolabs GmbH, Country) for DNA cloning and cell stock storage, and *E. coli* BL21(DE3)pLysS (New England Biolabs) as host strain for protein expression. For proper selection, ampicillin (Roche Diagnostics, Mannheim, Germany) (100 μg/ml) was used.

### Construction of plasmids

Inducible plasmid constructs for expression of either λSa2lys (NP_688827) or Art-240 were created using the pET21b backbone. Briefly, the sequence encoding λSa2lys was obtained as a synthetic construct from Thermo Fisher Scientific (Germany). The λSa2lys open reading frame was amplified with PCR primers (Table 2) comprising either a terminal NdeI or XhoI site to introduce appropriate restriction enzyme sites for sub-cloning into pET21b. The PCR product was gel purified, digested with NdeI and XhoI, purified using QIAquick PCR Purification Kit (QIAGEN GmbH, Germany) and introduced into similarly digested, dephosphorylated, and gel-purified pET21b via conventional means. Expression from the resultant pET21b/λSa2lys construct results in the addition of eight amino acid residues to the C-terminus of λSa2lys (corresponding to the XhoI site (Leu-Glu) followed by six His residues). The coding sequence for Art-240 was constructed by fusing the sequence encoding the polycationic nonapeptide (PCNP)^11^ to the 3′ end of the open reading frame of λSa2lys endolysin using a two-step standard PCR cloning method with PCR primer pairs: λSa2lysNdeIFw and λSa-His_6_AflII-P-XhoIRv-1 in a first PCR and λSa2lysNdeIFw and His6AflII-P-XhoIRv-2 in a second PCR using the product of the first PCR as a template (Table 2). The PCR amplified fragment (encoding Art-240) comprises subsequently the coding sequence for λSa2lys, a His_6_-tag for affinity purification, a unique AflII restriction site for flexible construction of different PNCP-based constructs and the PCNP-tag. The resulting λSa2lys-His_6_-AflII-PNCP encoding cassette was then introduced to modified pET21b, in which the C-terminal His_6_-tag was removed, via Quick Change mutagenesis (Q5 site-directed mutagenesis kit; New England BioLabs, GmbH) by introduction of two stop codons after the XhoI restriction endonuclease site. An internal His_6_-tag was preferred to ensure a free terminal PCNP.

### Recombinant expression and purification

The recombinant expression of both λSa2lys and Art-240 was performed in 2 liters lysogeny broth (LB) in *E. coli* BL21(DE3)pLysS and *E. coli* BL21(DE3) cells, respectively. Briefly, a preculture (50 ml LB in 100 ml flask inoculated from fresh plate) was grown overnight at 37 °C. The cells were inoculated into fresh medium at an optical density of OD_600_ = 0.1 and grown at 37 °C to an optical density of OD_600_ = 0.45–0.55. Next, the expression of the proteins was induced with 1 mM isopropyl-β-d-thiogalactopyranoside (IPTG) at 16 °C for 18 h. *E. coli* cells were harvested by centrifugation (6,000 × g for 5 min at 4 °C), and the obtained cell pellet was resuspended in 40 ml of lysis buffer (20 mM HEPES, 1 M NaCl, 20 mM imidazole and 1 mM MgCl_2_ [pH 7.4] supplemented with 25 μg/ml DNaseI). Cell disruption was done by sonication (Bandelin Sonopuls HD3200) under the following conditions: 45% maximum amplitude (51 watts) for 6 min with 10-s pulse/20-s break steps on ice. Cell debris was separated by centrifugation at 15,000 × g for 20 min, and the supernatant was then filtered (Filtropur S 0.2, PES-membrane, 0.2 μm pore size; Sarstedt AG & Co., Germany) before application to the column. Purification of the His_6_-tagged fusion proteins was performed on an AKTÄ fast protein liquid chromatography (FPLC) system (GE Healthcare, Little Chalfont, United Kingdom) controlled by UNICORN 5.1 software with Ni^2+^-charged immobilized-metal affinity chromatography (IMAC) columns (1 ml HiPrep IMAC FF; GE Healthcare). Bound proteins were washed using buffer A (20 mM HEPES, 1 M NaCl, 20 mM imidazole [pH 7.4]) and eluted with a linear gradient to 100% buffer B (20 mM HEPES, 500 mM NaCl, 500 mM imidazole [pH 7.4]). The fractions containing the purified λSa2lys endolysin or Art-240 were pooled and dialysed against buffer C (20 mM HEPES, 500 mM NaCl [pH 7.4]). After dialysis, the protein concentration was determined spectrophotometrically in silica cuvettes at a wavelength of 280 nm (Jasco V-650; Jasco Corporation, Tokyo, Japan).

### CD spectroscopy

To measure the melting temperature (Tm), the ellipticity of the proteins was recorded at 220 nm in a Jasco model J-815 circular dichroism (CD) spectrometer (Jasco Corporation). The protein melting temperatures were determined with a heating rate of 1 °C/min, an incubation time of 3 s, and a volume of 410 μl in a 1-mm light path Hellma quartz cuvette. All measurements were performed in 50 mM sodium phosphate buffer with 300 mM NaCl at pH 7.4. The midpoint of the unfolding transition was determined by fitting to a sigmoid unfolding model using JASCO analysis software. The proteins were measured at concentrations of 6.03 μM (λSa2lys endolysin) and 6.00 μM (Art-240).

### Muralytic assay

A standardized turbidity assay modified from Donovan *et al*.^6^ with *S. agalactiae* LiCC S461 as substrate was used. Briefly, *S. agalactiae* LiCC S461 cells in the mid-exponential phase (OD_600_ = 0.6) and stationary phase (OD_600_ = 1.2) were harvested by centrifugation (3,900 × g, 10 min, 4 °C) and stored on ice for ≤4 h until just before the assay. The cell pellets were suspended in 20 mM HEPES, 150 mM NaCl (pH 7.4) to an OD_600_ = ~1.2. In the experiment to study the effect of different pH values on the enzymatic activity of λSa2lys and Art-240, a universal buffer (150 mM KCl, 10 mM KH_2_PO_4_, 10 mM sodium citrate, 10 mM H_3_BO_3_) with different pH values between 4 and 10 was used in order to exclude any possible effect of the used buffer compound. Upon addition of 30 μl of muralytic enzymes (1 μM) to 270 μl of bacterial cells (final protein concentration 0.1 μM), the optical density was measured spectrophotometrically over time at 655 nm using a Microplate Reader 680 system (Bio-Rad). The standardized calculation method previously described^30^ was used to quantify the muralytic activity of the enzymes with one unit corresponding to a decrease in optical density of 0.001/min.

### Antibacterial activity

Bacterial cells at the mid-exponential growth phase (OD_600_ = 0.6) were harvested by centrifugation (16,000 × g for 5 min), washed, and diluted 100-fold in 20 mM HEPES 150 mM NaCl (pH 7.4) to a final density of ±10^7^ CFU/ml. Fifty μl of a protein solution containing 0.2 μM λSa2lys endolysin/Art-240 (or to the indicated concentration) in 20 mM HEPES, 150 mM NaCl (pH 7.4) was added to 50 μl of the bacterial cell dilution and further incubated at 25 °C, with shaking. After 60-min incubation, the reaction was stopped by addition of 0.15 μg Proteinase K and the appropriate dilutions of the cell suspensions were plated on BHI or LB agar in triplicate. Colonies were counted after overnight incubation at 37 °C. The antibacterial activity was quantified as the relative inactivation in log units (log_10_[*N*_*0*_/*N*_*i*_] with *N*_*0*_ as the initial number of untreated cells and *N*_*i*_ as the number of residual cells counted after treatment).

### Time–kill curve

Mid-exponentially growing cells (OD_600_ = 0.6) of *S. agalactiae* LiCC S461 in BHI medium were harvested by centrifugation (16,000 × g for 5 min), washed twice with 1 ml 20 mM HEPES, 150 mM NaCl (pH 7.4), and resuspended in 1 ml 20 mM HEPES, 150 mM NaCl (pH 7.4) to a final density of ±10^7^ CFU/ml. A suspension with a total volume of l ml containing 950 μl of the bacterial cell dilution, and 50 μl λSa2lys endolysin/Art-240 in 20 mM HEPES, 500 mM NaCl (pH 7.4) (final concentration 0.1 μM) was prepared. These mixtures were incubated at 25 °C in a shaker for the times indicated. At the respective time points, 50 μl was taken, the reaction was stopped with 0.15 μg Proteinase K, and appropriate dilutions of the cell suspensions were plated on BHI agar in triplicate. Colonies were counted after overnight incubation at 37 °C. The antibacterial activity was quantified as indicated above.

### Plate lysis assay

Purified λSa2lys/Art-240 were diluted in sterile 20 mM HEPES, 500 mM NaCl (pH 7.4) buffer, and 5 μl from a stock solution of 5 μM (1.25 μg), 2 μM (0.5 μg) and 1 μM (0.25 μg) was spotted onto BHI or LB agar plate overlaid with 3 ml 0.75% BHI or LB soft agar containing 100 μl the target bacteria from an overnight culture. The spotted plates were air dried for 10 min under a laminar flow hood and incubated overnight in a 37 °C environment. Semi-quantitative scoring of the cleared spots occurred after 24 h of plating the cells.

### Time-lapse microscopy

Exponentially growing *S. uberis* LiCC S648 cells (37 °C, BHI, OD_600nm_ = 0.6) were washed three times in 20 mM HEPES, 500 mM NaCl pH 7.4 and concentrated five times in the same buffer. Equimolar amounts of Art-240, λSa2lys and the PCNP (0.2 μM) in 20 mM HEPES, 500 mM NaCl pH 7.4 were mixed in a 1:1 ratio with the cell suspension (final concentration of compounds is 0.1 μM), and the mixture was immediately transferred to an agarose pad (2% dissolved in 20 mM HEPES, 500 mM NaCl [pH 7.4]) for imaging. A buffer control (20 mM HEPES, 500 mM NaCl [pH 7.4]) was included. Time-lapse microscopy experiments were performed with a temperature controlled (Okolab, Ottaviano, Italy) Eclipse Ti-E inverted microscope (Nikon, Champigny-sur-Marne, France) equipped with a Ti-CT-E motorized condenser and a CoolSNAP HQ2 FireWire CCD camera, as described previously^31^. Images were acquired using NIS-Elements (Nikon), and the resulting pictures were further handled with open-source software, ImageJ (http://rsbweb.nih.gov/ij/).

### Statistical analyses

The one-tailed Student *t*-test was used to compare the antibacterial activity of λSa2lys and Art-240 (expressed as log viable reduction) against a panel of strains. The same test was used to compare the effect of pH and NaCl on the enzymatic activity of both proteins against exponential cultures of *S. agalactiae* LiCC S461 and *S. uberis* LiCC S648, as well as their activity against exponential and stationary cultures of *S. agalactiae* LiCC S461 (expressed as the slope of the killing curves). The killing effect of the enzymes over time (0–30 min) and at different concentrations (6.25–200 nM) against *S. agalactiae* LiCC S461 suspensions at pH 7.4 were also compared (expressed as log viable reduction). Results are shown as means ± standard deviations. A *P* value below 0.05 was considered statistically significant.

## Additional Information

**How to cite this article**: Rodríguez-Rubio, L. *et al*. ‘Artilysation’ of endolysin λSa2lys strongly improves its enzymatic and antibacterial activity against streptococci. *Sci. Rep.* **6**, 35382; doi: 10.1038/srep35382 (2016).

## Supplementary Material

Supplementary Information

Supplementary Movie S1

## Figures and Tables

**Table 1:** Spectrum of activity for endolysin λSa2lys and Art-240.

Bacteria	Strain	LiCC Nr.	λSa2lys			Art-240			PNCP	Buffer
			5 μM	2 μM	1 μM	5 μM	2 μM	1 μM	10μM	
***Streptococcus***										
*S. agalactiae*	B12-11.20.0190	S439	++	+	+/−	++	+	+/−	—	—
	vaOO413	S461	++	++	+	+++	+++	++	—	—
	va80306	S1047	++	++	+	+++	+++	+	—	—
	1560	S1046	+	+	+/−	++	+	+/−	—	—
*S. dysgalactiae*	B12-11.14.0245	S436	+++	++	++	+++	+++	++	—	—
*S. pyogenes*	DSMZ 11728	S271	++	++	++	+++	+++	+++	—	—
	3347	S328	+++	+++	+++	+++	+++	+++	—	—
	10651	S1049	+++	+++	+++	+++	+++	+++	—	—
*S. uberis*	C2638	S636	++	+	+	+++	++	+	—	—
	C2673	S637	+	+	+	++	++	++	—	—
	C2623	S639	+	+	+	++	+	+	—	—
	C2691	S640	+++	+++	++	+++	+++	++	—	—
	C2697	S641	++	+	+	+++	++	++	—	—
	C2746	S644	+++	++	++	+++	+++	++	—	—
	C2761	S646	+	+/−	—	++	+	+/−	—	—
	C2795	S648	+++	++	+	+++	+++	++	—	—
	C2811	S649	++	+	+/−	+++	++	+	—	—
	C2813	S650	+++	+++	++	+++	+++	+++	—	—
	C2818	S652	+++	+	—	+++	++	+	—	—
	C2819	S653	++	+	—	+++	++	+	—	—
	C2814	S654	+	+/−	—	++	+	+/−	—	—
*S. suis*	SN 16770	S772	+/−	—	—	+	+/−	—	—	—
	SN 19395	S774	+	+	+/−	++	+	+	—	—
	SN 19396	S775	++	+	+	++	++	+	—	—
	SN 20014	S777	+	+/−	+/−	++	+	+	—	—
	SN 23674	S778	+	+/−	+/−	+	+/−	+/−	—	—
	SN 26556	S779	+	—	—	+	+/−	—	—	—
	SN 29429	S780	+	+/−	—	++	+	+/−	—	—
*S. porcinus*	2231	S1048	+/−	+/−	—	+	+	+/−	—	—
	341/11/13	S848	+/−	—	—	+	+/−	—	—	—
*S. gordonii*	6365	S1050	+	+	+/−	++	+	+/−	—	—
*S. sanguinis*	10505	S1053	+	+	+/−	++	+	+/−	—	—
*S. viridans*	7351	S1054	++	++	+	+++	++	+/−	—	—
***Staphylococcus***										
*S. aureus*	DSMZ 346	S20	—	—	—	—	—	—	—	—
	DSMZ 20231	S21	—	—	—	—	—	—	—	—
	Sp10	S165	—	—	—	—	—	—	—	—
	Sp10res	S416	—	—	—	—	—	—	—	—
*S. epidermidis*	B12-11.20.0190	S440	+	+/−	—	++	+	+	—	—
***Enterococcus***										
*E.faecium*	NCIMB 11181	S526	—	—	—	—	—	—	—	—
***Pseudomonas***										
*P. aeruginosa*	PAO1p	S52	—	—	—	—	—	—	—	—
***Escherichia***										
*E.coli*	06-08410 **(O103:H2)**	S68	—	—	—	—	—	—	—	—
	03-07953 **(O157:H7)**	S72	—	—	—	—	—	—	—	—
***Salmonella***										
*S.* Hadar	LGL-107 **(Group C2-C3)**	S796	—	—	—	—	—	—	—	—
*S.* Typhimurium	LGL-57 **(Group B)**	S808	—	—	—	—	—	—	—	—

Plate lysis assay with thirty-three *Streptococcus* sp., five *Staphylococcus* sp. and one *Enterococcus faecium* bacterial strains as well as three Gram-negative bacterial strains (*E. coli, Pseudomonas aeruginosa, Salmonella* sp.). Lysis zones were analyzed in a semi-quantitative manner, with — corresponding to no visible lysis, and +/−, +, ++, +++ corresponding to an increasingly clear lysis determined in pairwise comparisons. Controls: buffer, PNCP peptide (10 μM spotted); Abbreviations: PCNP = polycationic nonapeptide.

+++ very good, ++ good, +low, +/−detectable, —no activity.

**Table 2:**
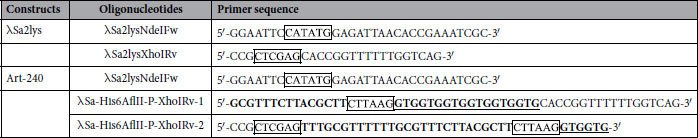
Overview of oligonucleotides.

The list of primers for PCR amplification used in this study are summarized. The restriction sites used for cloning are boxed in the respective primer sequence. Nucleotides encoding for the polycationic nonapeptide (PNCP) are indicated in bold. Nucleotides encoding the His6-tag are underlined and in bold.
