# Supplementary material for: ‘Artilysation’ of endolysin λSa2lys strongly improves its enzymatic and antibacterial activity against streptococci
Source: Sci Rep. 2016 Oct 24;6:35382. doi: 10.1038/srep35382 (PMC5075790; doi:10.1038/srep35382)
Supplement: Supplementary Information [file srep35382-s1.pdf]

Supplementary Material

'Artilysation' of endolysin λSa2lys strongly improves its enzymatic and antibacterial activity against streptococci

Lorena Rodríguez-Rubio[1,2†], Wai-Ling Chang[3†], Diana Gutiérrez[1], Rob Lavigne[2], Beatriz Martínez[1], Ana Rodríguez[1], Sander Govers[4], Abram Aertsen[4], Christine Hirl[3], Manfred Biebl[3], Yves Briers[5*] and Pilar García[1*].

*Corresponding authors; †Contributed equally

Supplementary Figures

Supplementary file 1. Purification and stability of λSa2lys endolysin and its derivative Artilysin, Art-240. Electrophoretic profile of proteins A) endolysin λSa2lys and B) Art-240. Lane 1, PeqLab Protein Marker I standard; lane 2, total fraction; lane 3, cytoplasmic fraction; lane 4, column flow through and lane 5 purified protein. C) Melting curves of both λSa2lys endolysin (triangle) and Art-240 (circle) performed by monitoring loss of α-helicity of proteins by CD spectroscopy at 222 nm. Cooperative melting is observed for each protein in a similar way. Tm values are 63.9°C and 61.1°C for Art-240 and λSa2lys, respectively.

A)

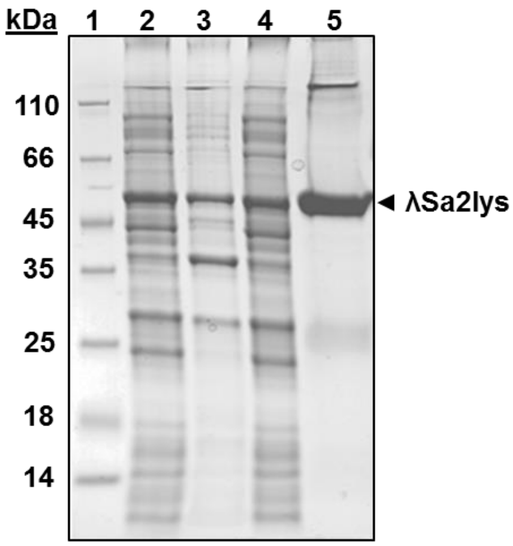

B)

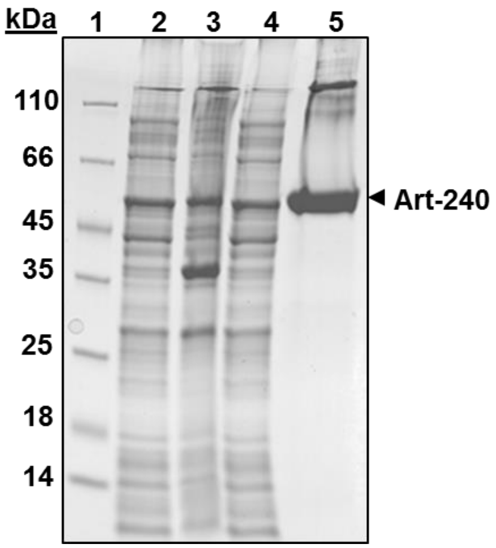

C)

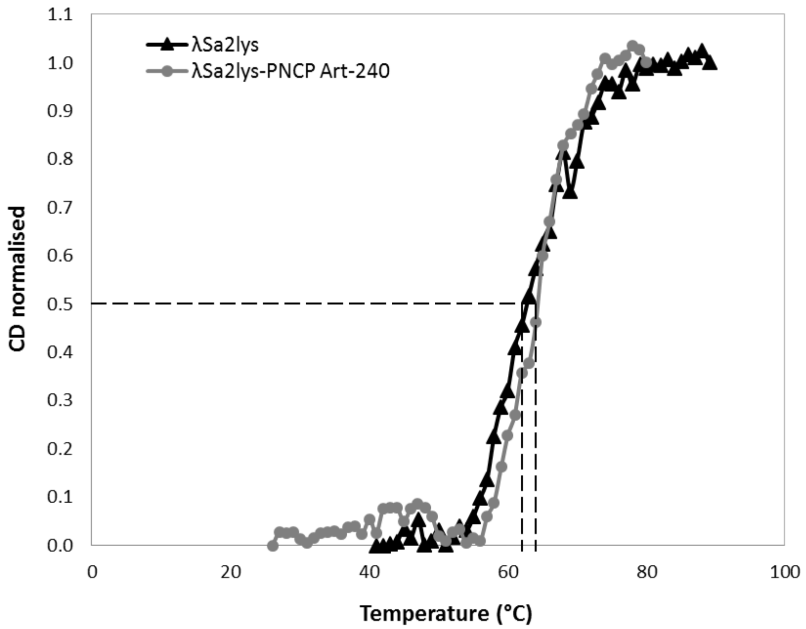

Supplementary file 2. Plate lysis assay of endolysin λSa2lys and Art-240 against *S. agalactiae* LiCC S461. Spots were dropped with 1, 2, and 5 µM of each protein. Control spots are the stock buffer of λSa2lys and Art-240 (20 mM HEPES 500 mM NaCl pH 7.4). +++ very high, ++ high, + moderate, +/- low, --- no activity.

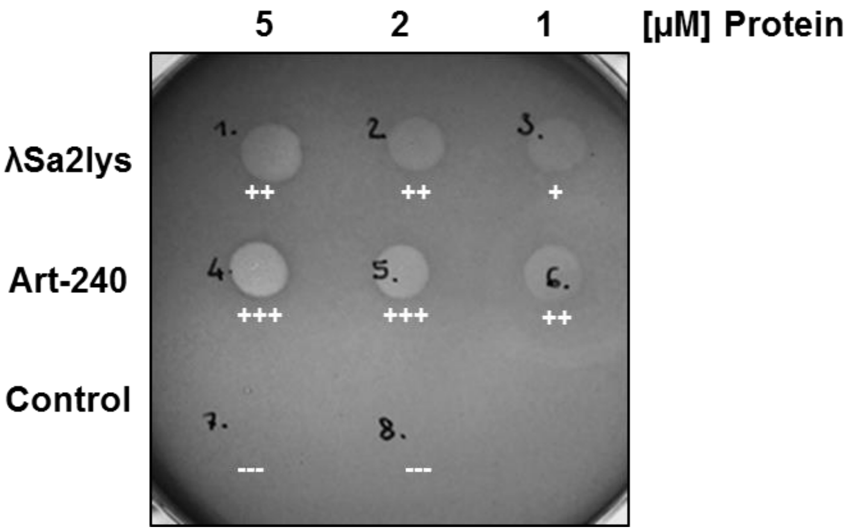

Supplementary file 3. Effect of pH on enzymatic activity of λSa2lys endolysin and Art-240. The activity was determined by a turbidity reduction assay with equimolar amounts (0.1 µM) of λSa2lys (grey) and Art-240 (white), and reported as the slopes derived from a 60-min curves from both *S. uberis* LiCC S648 performed under various pH conditions. Data are means ± standard deviations of three replicated. Student *t*-test was performed to compare the activity of λSa2lys and Art-240 within each pH value. *P*<0.01; *P*<0.001.

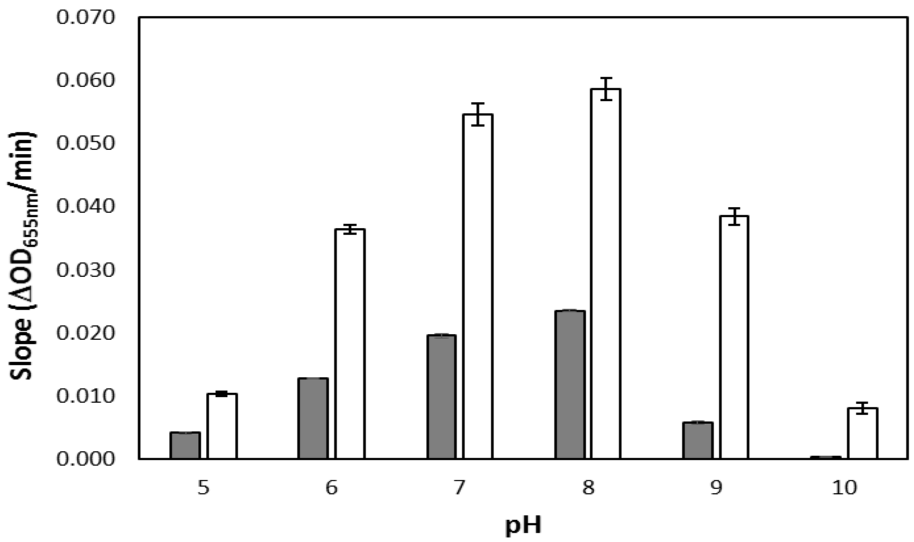

Supplementary file 4. Effect of NaCl on enzymatic activity of λSa2lys endolysin and Art-240. The activity was determined by using a turbidity reduction assay with equimolar amounts (0.1 µM) of λSa2lys (grey) and Art-240 (white), and reported as the slopes derived from a 60-min curves from *S. uberis* LiCC S648 performed under various sodium chloride (NaCl) conditions. Data are means ± standard of three replicated. Student *t*-test was performed to compare the activity of λSa2lys and Art-240 within each NaCl concentration. *P*<0.01; *P*<0.001.

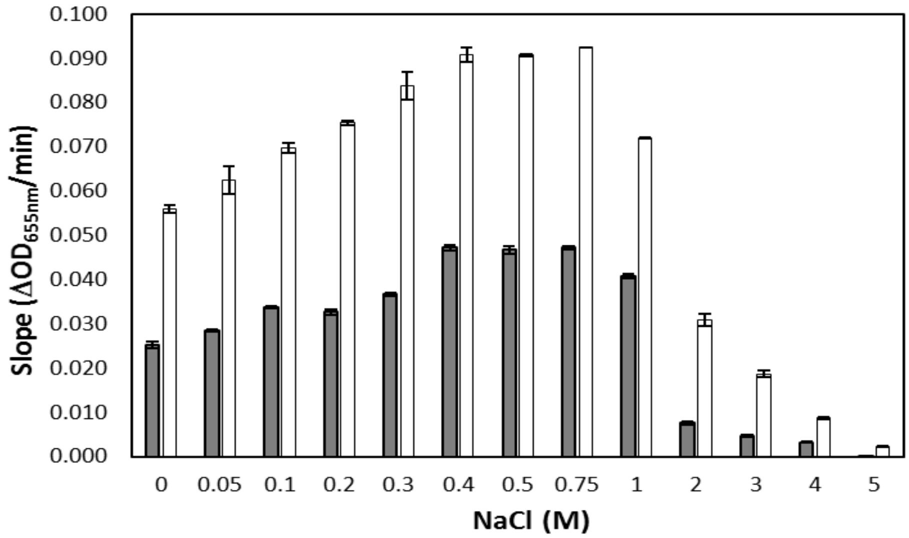

Movie S1. Real-time movie of *S. uberis* LiCC S648 exposed to equimolar amounts (0.1 µM) of Art-240, λSa2lys, PCNP and buffer. Exponentially growing cells were washed three times with buffer and subsequently mixed (1:1) with 0.2 µM of the corresponding enzyme/peptide. The mixture was dropped on agarose pads and cells were monitored in real-time.
